# From prognostication to implementation: the next frontier for ultrasound-guided assessment of decongestion

**DOI:** 10.1093/ehjimp/qyag140

**Published:** 2026-08-18

**Authors:** Abhilash Koratala, Amir Kazory

**Affiliations:** Division of Nephrology, Medical College of Wisconsin, 8701 W Watertown Plank Rd, Milwaukee, Wisconsin 53226, USA; Division of Nephrology, Hypertension and Renal Transplantation, University of Florida, Gainesville, Florida 32608, USA

**Keywords:** congestion, heart failure, VExUS, point-of-care ultrasound, training, lung ultrasound

To the editor,

We congratulate Silvano *et al*. on their elegant study demonstrating that ΔVExPLUs, a metric integrating serial changes in the venous excess ultrasound score (VExUS) and lung ultrasound (LUS), is independently associated with short-term outcomes in patients hospitalized with acute decompensated heart failure (ADHF).^1^ This work builds upon the growing body of evidence supporting the utility of bedside ultrasound in heart failure. Recent studies have established that residual venous congestion identified by VExUS at hospital discharge predicts long-term mortality and heart failure rehospitalization.^2^ Of note, the present study offers a more comprehensive physiologic assessment of the evolving congestive state by integrating systemic venous (right-sided) and pulmonary (left-sided) congestion. We believe a key contribution of this study is not merely demonstrating another prognostic marker but reframing multiorgan ultrasound as a dynamic physiologic biomarker that can be repeatedly measured to monitor response to therapy. This conceptual shift is particularly important because, although congestion has long been recognized as a major therapeutic target in heart failure, comparatively less attention has been devoted to developing practical bedside methods to objectively quantify and monitor the very process we seek to treat.

The next challenge is no longer prognostic validation but real-life implementation. If serial ultrasound assessment of congestion is to become part of routine heart failure care, who should perform these examinations, interpret them, and incorporate their findings into day-to-day clinical management? The ultrasound examinations in this study were performed by cardiology fellows who completed a structured institutional training programme. Their background in echocardiography and Doppler ultrasound likely provided a strong foundation for VExUS, an application that demands greater technical expertise than routine point-of-care ultrasound examinations.^3^ As ultrasound-guided congestion assessment moves beyond the cardiac intensive care unit into routine heart failure care, where multiple disciplines share responsibility for longitudinal congestion management, clinicians will bring widely varying levels of ultrasound experience.

Each discipline brings unique strengths. Cardiologists and cardiac sonographers are highly skilled in echocardiography but may have limited experience with VExUS and LUS. Abdominal sonographers routinely evaluate the hepatic and portal veins but generally do not perform integrated haemodynamic assessments, electrocardiographically gated venous Doppler, or LUS. Radiologists provide expert interpretation of abdominal Doppler examinations but are not typically tasked with integrating these findings with echocardiographic physiology to distinguish congestion amenable to volume removal from Doppler abnormalities related to atrial fibrillation, right ventricular dysfunction, or severe tricuspid regurgitation. Clinicians trained in point-of-care ultrasound are uniquely positioned to acquire multiorgan ultrasound findings and integrate them with the evolving bedside clinical picture; however, training pathways and expectations remain heterogeneous across specialties. Greater educational standardization will be essential if the promising results demonstrated in research settings are to translate into meaningful improvements in patient care. Emerging artificial intelligence (AI)-assisted tools may guide novice users during image acquisition, automate image labelling and VExUS scoring, and reduce operator variability. Nevertheless, AI should complement rather than replace clinician training, as accurate interpretation and integration with the patient’s clinical context remain critical. *Figure 1* summarizes the proposed roadmap for translating VExUS from research into routine clinical practice.

**Figure 1:**
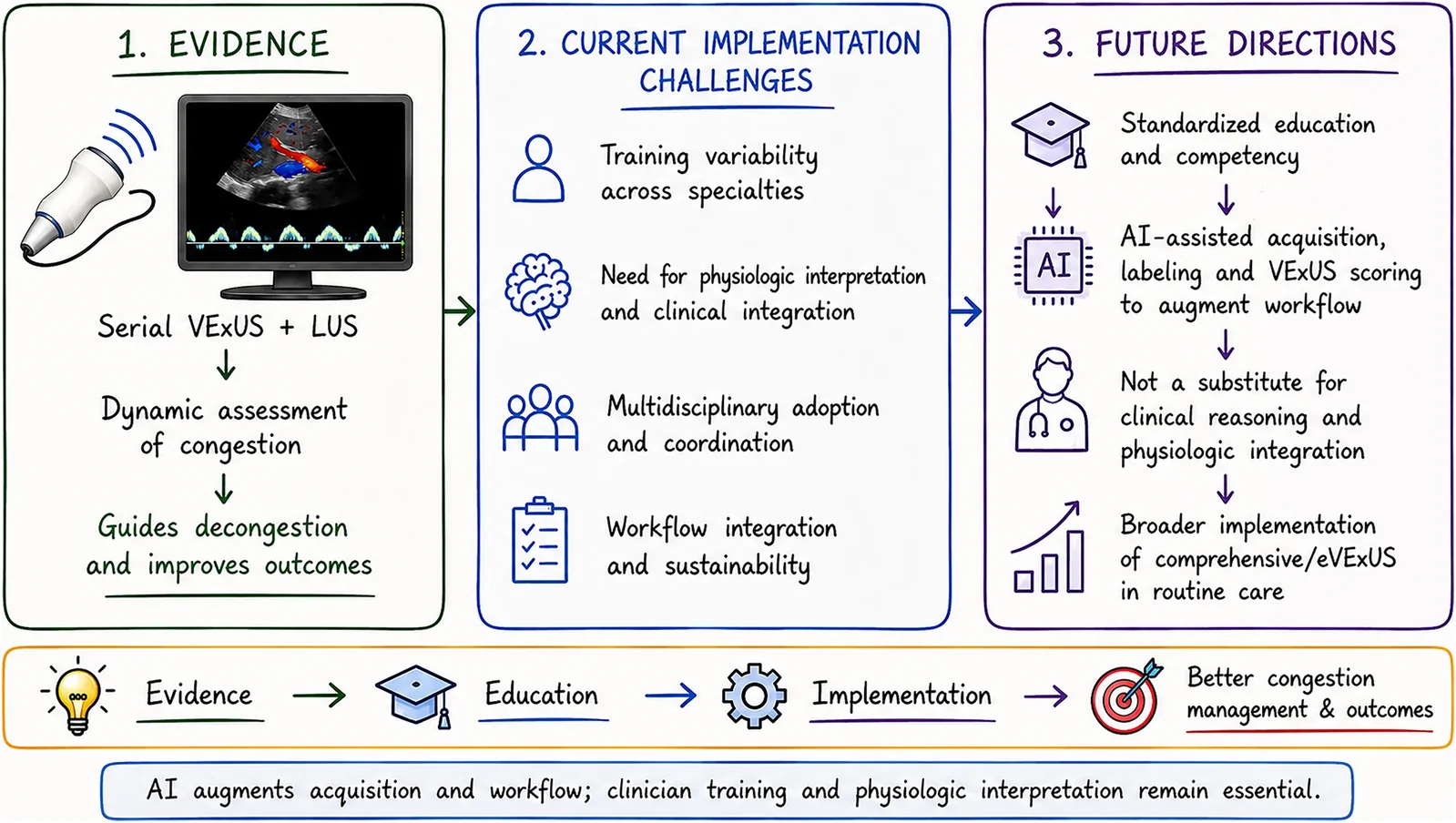
Conceptual framework for translating VExUS from research into clinical practice. Current evidence supports serial VExUS combined with LUS for objective, dynamic assessment of congestion. Broader implementation will require standardized education, competency-based training, multidisciplinary integration, and workflow adaptation. Evolving AI tools may facilitate workflow but should complement, not replace, clinician training, physiologic interpretation, and clinical integration.

The modified VExUS protocol adopted in this study, which excluded intrarenal venous Doppler, appropriately prioritizes feasibility for serial bedside assessment in ADHF. Nevertheless, intrarenal venous Doppler has been well studied in this patient population and provides complementary information, particularly regarding renal congestion and kidney-related outcomes. Therefore, while simplified protocols may be appropriate in selected clinical settings, they should not be interpreted as diminishing the broader relevance of intrarenal venous Doppler. VExUS is increasingly being applied beyond ADHF to cardiorenal syndromes, critical illness, cirrhosis, pulmonary hypertension, end-stage kidney disease, and perioperative medicine, where both the physiologic limitations and technical feasibility of individual venous Doppler components may differ substantially. Some centres have adopted modified VExUS protocols that substitute femoral vein Doppler for intrarenal venous Doppler, whereas others have proposed extended VExUS approaches incorporating additional venous territories to improve feasibility or provide complementary haemodynamic information.^4,5^ Although abbreviated protocols may improve feasibility in selected settings, they should be viewed as pragmatic entry points rather than specialty-specific alternatives. The physiologic principles underlying venous congestion remain the same across disciplines, and VExUS should therefore be adapted to the clinical context rather than the clinician’s specialty. Clinicians responsible for longitudinal management should understand the comprehensive haemodynamic framework, as isolated assessment of a single venous territory may provide an incomplete picture and fail to identify the most appropriate therapeutic strategy.

Ultimately, heart failure care has always been multidisciplinary, and ultrasound-guided congestion assessment should advance in the same collaborative spirit. As evidence continues to grow, professional societies across cardiology, nephrology, critical care, internal medicine, and advanced practice nursing should work together to develop standardized curricula, competency-based training, quality assurance frameworks, and multidisciplinary educational pathways. Such efforts should emphasize not only how these examinations are performed but also the physiologic principles that enable clinicians to thoughtfully adapt them to different patient populations as the evidence and techniques continue to evolve. Without parallel investment in education and implementation, these advances risk remaining confined to research rather than transforming patient care. Given the rapid pace of the field, establishing the necessary educational infrastructure is not merely desirable but urgent. Ideally, clinicians making day-to-day haemodynamic decisions should acquire competency in VExUS because point-of-care ultrasound is fundamentally clinician-performed. Even in practice settings where image acquisition and interpretation are performed by dedicated ultrasound experts, treating clinicians should understand the physiologic implications and limitations of the sonographic findings so they can appropriately integrate them into patient management.

## Data Availability

No new data were generated or analysed in support of this research.

## Lead author biography

Abhilash Koratala, MD, FASN, FNKF, is a nephrologist and intensivist who serves as an Associate Professor at the Medical College of Wisconsin. He is board certified in critical care echocardiography and holds National Board of Echocardiography testamur status in advanced perioperative transoesophageal echocardiography. Dr Koratala is internationally recognized for his work in point-of-care ultrasound (POCUS), particularly in Doppler-based bedside haemodynamic assessment. He has delivered over 150 invited lectures worldwide and is an active medical educator on Twitter/X as @NephroP, where he engages over 100,000 learners.

His educational platform, NephroPOCUS.com, received the American Society of Nephrology (ASN) Innovations in Kidney Education Award in 2020, and he was honoured by ASN as a Distinguished Educator in 2024, one of the society’s most prestigious recognitions for excellence in teaching. His scholarly work includes more than 250 peer-reviewed publications.
